# *Nesticus baeticus* sp. n., a new troglobitic spider species from south-west Europe (Araneae, Nesticidae)

**DOI:** 10.3897/zookeys.89.921

**Published:** 2011-04-11

**Authors:** Alberto López-Pancorbo, Carles Ribera

**Affiliations:** *Institut de Recerca de la Biodiversitat & Departament de Biologia Animal. Universitat de Barcelona, Av. Diagonal, 645, Barcelona - 08028, Spaina*

**Keywords:** Arachnida, Araneae, taxonomy, description, new species, caves, Iberian Peninsula, Mediterranean basin

## Abstract

A new troglobitic species, Nesticus baeticus **sp. n.** (♂♀), inhabiting the karst landscapes of the high part of the Cazorla, Segura and Las Villas Natural Park (NE Jaén, Spain) where it has been found in 8 caves is diagnosed and described, its distribution and habitat are also analyzed.The new species belongs to the Iberian species group that includes Nesticus luquei, Nesticus lusitanicus and Nesticus murgis. Evolutionary relationships of the Iberian Nesticus species are discussed on the basis of morphological and molecular data (*cox1* and *rrnL*).

## Introduction

The genus Nesticus Thorell, 1869 is distributed worldwide except for south-eastern Asia and Australia and comprises 125 species and 8 subspecies (Platnick 2011). In Europe Nesticus is represented by 23 species, of which five are known from Iberia. Unlike Nesticus cellulanus (Clerck, 1757), a species with a holarctic distribution, the four others are endemic to Iberia, being cavernicolous species with more or less evident troglomorphic features.

The first species described from Iberia was Nesticus obcaecatus Simon, 1907, found only from a single locality: Cueva del Molino de Aso (Huesca), on the southern slopes of the Central Pyrenees (Simon 1907). The species description was originally based on a single female specimen. The male was described several decades later (Ribera 1979), from a series of both sexes collected from the same cave. The second Iberian endemic is Nesticus lusitanicus Fage, 1931, a native species of the karst landscape in central Portugal. This species was described on the basis of females (Fage 1931), its male was found half of a century later (Ribera 1988). The third species, which was found in several caves in Asturias and Cantabria (north-western Iberian Peninsula) (Ribera and Guerao, 1995), is Nesticus luquei Ribera & Guerao, 1995. Finally, Nesticus murgis Ribera & De Mas, 2003 was described from a cave in the province of Almeria (Ribera and De Mas 2003). Overall, ranges of these species are rather small, in two species (Nesticus obcaecatus and Nesticus murgis) being restricted to a single cave.

This work describes a new cavernicolous species whose distribution includes several karst landscapes of the different mountain formations that make up the Sistema Bético, the ridge in southern Iberian Peninsula.

## Materials and methods

### Taxonomy

Abbreviations used in the text are as follows: PL = prosoma length (from posterior edge of carapace to front edge of clypeus, measured at midline); PW = maximum prosoma width; OL = opisthosoma length (excluding the pedicel); OW = maximum opisthosoma width; MA = median apophysis; Mt = metatarsus; Tb = tibia; TTA = theridioid tegular apophysis; TTA p1 = process 1 of TTA; TTA p2 = process 2 of TTA; TL = total length (excluding the pedicel). Eyes: AM = anterior median; AL = anterior lateral; PM = posterior median; PL = posterior lateral; i = immature; sub = subadult; GEV = Grupo de Espeleología de Villacarrillo.

Female vulva was removed and treated with 30% KOH. After observation and drawing, the vulva was washed in distilled water and stored in 70% ethanol. Left male palps were illustrated in all cases. We follow Coyle and McGarity (1992) for describing the paracymbium, and Huber (1993) and Agnarsson et al. (2007) for other parts of male and female copulatory organs. Holotype and paratypes have been deposited in the Arachnida Collection of the CRBA (Centre de Recursos de Biodiversitat Animal) at the University of Barcelona. Catalogue numbers are given in brackets.

A Nikon Coolpix 4500 digital camera attached to a stereomicroscope was used to capture images. Following Coleman (2003) and with the aid of a Trust Scroll Tablet TB-4200 the samples were drawn with repeated reference to the specimen under the microscope. The specimens used for SEM studies were dehydrated with alcohol gradient dehydration and ultrasonically cleaned. They were then critical-point dried and were mounted and covered with gold and examined using a HITACHI S-2300 Scanning Electron Microscope (SEM) (SCT, Universitat de Barcelona, Spain).

### Phylogeny

#### Taxonomic sampling.

Taxa analyzed in the present study are listed in Appendix 1. All the Iberian species are included except Nesticus murgis due to impossibility to obtain fresh material for DNA analysis. Nesticus eremita Simon, 1879 from Croatia and Nesticus ionescui Dumitrescu, 1979 from Romania are also included to test the monophyly of Iberian species. Sequence from Nesticus sp. from China (Arnedo et al. 2004) was also included in the analysis as a more distantly related Nesticus species that was used to root the tree.

#### Sample Storage and DNA Extraction.

Specimens were preserved in 95% or absolute ethanol and stored at 4°C. Total genomic DNA was extracted from legs of a single specimen using the QIamp® DNA Mini Kit (QIAGEN) following the manufacturer’s protocols. The approximate concentration and purity of the DNA obtained were verified using 1% agarose/TBE gel electrophoresis.

#### PCR Amplification and Sequencing.

Two regions of the mitochondrial DNA corresponding to a fragment of the cytochrome oxidase I gene (*cox1*) and 16S rRNA (*rrnL*) were selectively amplified using PCR with the following primer pairs: for *cox1* C1-J-1718 (5' GGAGGATTTGGAAATTGATTAGTTCC 3') with C1-N-2191 (5' CCCGGTAAAATTAAAATATAAACTTC 3') (Simon et al. 1994); for *rrnL* LR-N-13398 (5' CGCCTGTTTATCAAAAACAT 3') (Simon et al. 1994) with LR-J-12864 (5' CTCCGGTTTGAACTCAGATCA 3') (Arnedo and Gillespie 2006). The PCR reaction mixture contained a final concentration of 0.2 μM of each primer, 0.2 mM of each dNTPs, 0.5 U Taq polymerase (Promega), with the supplied buffer, and 1.5–2.5 mM Mg Cl2 in a final volume of 25 μL.

A Perkin-ElmerCetus Moldel 480 thermocycler was used to perform 35 iterations of the following cycle: 30s at 95°C, 45s at 45°C, and 1 min at 72°C, beginning with an additional step of 3 min at 95°C, and ending with another step of 10 min at 72°C. PCR results were visualized by means of a 1% agarose/TBE gel. Amplified products were purified using Microcon PCR columns following the manufacturer’s specifications. Purified products were directly cycle-sequenced from both strands using ABI BigDye (Applied Biosystems) chemistry, precipitated in DyeEx Spin kit (Qiagen, Chatsworth, CA) columns, and run out on ABI Prism 377 (Applied Biosystems) automated sequencers. Sequencing reactions were performed in our lab with the forward and reverse PCR primers. Resulting product were run and analyzed at the Serveis Científico-Tècnics of the Universitat de Barcelona.

#### Alignment.

Raw sequences were compared against chromatograms and complementary contigs built and edited using the Geneious Pro 3.6.2 software (http://www.genious.com). Sequences were manipulated and preliminary manual alignments constructed using BioEdit V.7.0.5.3 (Hall 1999). Alignment of the *cox1* gene fragments was trivial due to the absence of length polymorphism. However, there were some length differences among the *rrnL* fragments, suggesting the occurrence of insertion/deletion events during the evolution of these sequences. Automatic alignment algorithms have been considered as superior to manual protocols due to their objectivity and repeatability (Giribet et al. 2002). Automatic alignments for the *rrnL* data set were constructed with the program MAFFT v 6.240 (Katoh and Toh 2007) The alignment was constructed using the manual strategy option set Q-INS-I, the most accurate multiple sequence alignment, whit default options. All analyses were performed by coding gaps as absence/presence character following Simmons and Ochoterena’s simple coding method (Simmons and Ochoterena 2000), as implemented in the software GAPCODER (Young and Healy 2002). This method allows the inclusion of gap information in phylogenetic inference, minimizing the effect of increasing the weight of overlapping multiple non-homologous gaps that results from scoring gaps as 5th state (Pons and Vogler 2006).

#### Phylogenetic analyses.

Parsimony analyses of the combined data matrices were conducted with the program Winclada v.1.00.08 (Nixon 2002) using the following heuristic tree search strategy: 1000 iterations of 10 Wagner trees constructed with random addition taxa and subsequent TBR branch swapping, holding a total maximum of 10000 trees. This program facilitated combination of the different gene fragments in a single data set for simultaneous analyses and also provided additional statistics for those trees (CI and RI values). Clade support was assessed via Bootstrap (Felsenstein 1985) as implemented in Winclada, based on 1000 bootstrap replicates with 20 iterations and 10 starting trees per replica. Uncorrected genetic distances between taxa of *cox 1* gene from terminal taxa were assessed with the program MEGA v.3.0 (Kumar et al. 2004).

## Description

### Nesticus baeticus sp. n.

urn:lsid:zoobank.org:act:51EE521C-466B-47F7-81F6-244EF9FA8547

**Figure 1.**
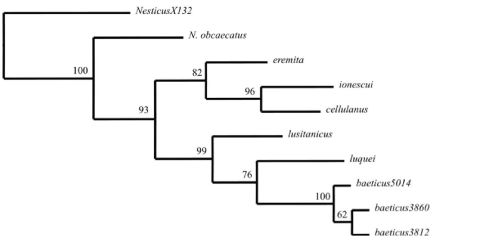
Single most parsimonious tree (L = 3454, CI = 0.447, RI = 0.769) found by MP analysis of the combined data set(*cox1*=472 bp, *rrnL*=456 bp and 22 gap characters) of Iberian Nesticus species (except Nesticus murgis), Nesticus ionescui from Romania and Nesticus eremita from Croatia. Numbers on nodes represent bootstrap support values. The outgroup Nesticus X130 is from China. Numbers on terminals refers to different localities (see material examined)

Figs 1 [Fig F2] [Fig F3] 17 

#### Material examined.

**Holotype:** ♂ (1619-A25) Cueva de la Murcielaguina, Hornos, Jaén, Spain, 5.11.2006, GEV leg. **Paratypes:** 2♀♀ (1619-A25) same locality and data; 1♀ (1720-A29) same locality, 30.12.2007, GEV leg.; 1♀ (1530-A22) Sima HO-55, Hornos, Jaén, 14.8.2006, GEV leg. (drawings and description of the female are based on this specimen); 1♂ (1524-A21), 1♂sub., 1♀, 7i (1525-A22) same locality and data, GEV Leg.; 1♀ (3811–150) Sima de los Alhaurinos, Hornos, Jaén, 12.05.2002, GEV leg.; 1♀ (3812–150) same locality and data; 2i (3860-151) Sima del Campamento, Hornos, Jaén, 02.03.2003, GEV leg.; 2i, (5023-189) same locality, 27.08.2004, GEV leg.; 2♂sub., 1f, 5i (1157-A07) Sima del Laberinto, Hornos, Jaén, 04.02.2006 López, A. & Pérez, A. leg.; 1♀, 1♀sub. (1343-A14) Cueva SE-20, Santiago de la Espada - Pontones, Jaén, 30.04.2006, GEV leg.; 1i (1539-A22) Sima del Órgano (HO-25), Hornos, Jaén, 14.08.2006, GEV leg.; 1i (5014-189) Sima Irene, Hornos, Jaén, 15.02.2004, GEV leg.

#### Etymology.

The Latin name ‘*baeticus’* means ‘from Baetica’ (the south of Spain) and refers to the ‘Sistema Bético’, the ridge containing the karst landscapes from where the new species was collected.

#### Diagnosis.

Males clearly differ from those of other Nesticus species in the shape of paracymbium (Figs 4–5, 8–10) and in the TTA structure (Figs 3–5, 11–12). In females, the development of the median septum of the vulva (Figs 14–16), the shape of the spermathecae and adjoining structures are also diagnostic (Fig. 17). The degree of ocular reduction of the AM eyes (Fig. 2) is also characteristic compared to other Iberian species.

**Figures 2–7. F2:**
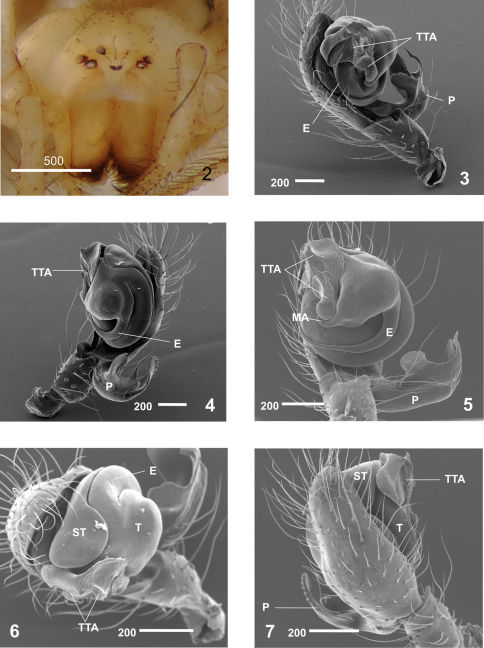
Nesticus baeticus sp. n.: **2** female (1525) frontal view **3–6** male palp (Holotype). **3** retrolateral view **4** prolateral view **5** ventral view **6** frontal view **7** dorsal view. Abbreviations: **E** embolus **MA** median apophysis **P** paracymbium **ST** subtegulum **T** tegulum **TTA** theridioid tegular apophysis. Scale bar in μm.

#### Comments.

**Figures 8–13. F3:**
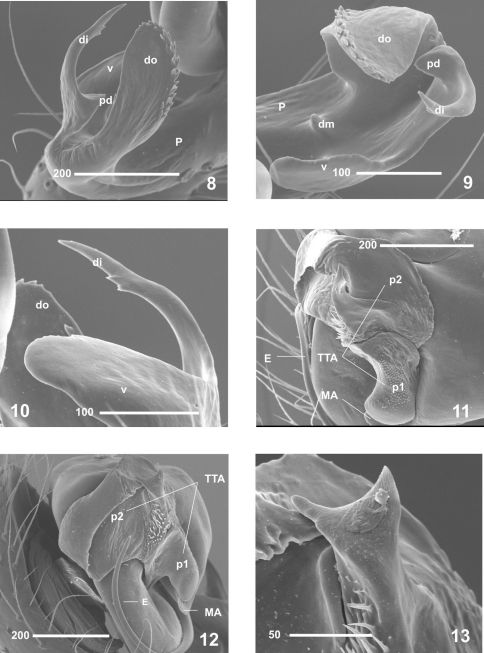
Nesticus baeticus sp. n., male (holotype). **8–10** paracymbium **8** lateral view **9** frontal view **10** ventral view **11** median apophysis and theridioid tegular apophysis, ventral view **12** ditto, retrolateral view **13** apical protuberance of TTA p2. Abbreviations: **di** distal process **dm** dorsomedial apophysis **do** dorsal process **E** embolus **P** paracymbium **pd** paradistal apophysis **MA** median apophysis **TTA** theridioid tegular apophysis **TTA p1** process 1 of TTA **TTA p2** process 2 of TTA **v** ventral process. Sacale bar in μm.

On the basis of morphology, Nesticus baeticus sp. n. lies within the group including Nesticus murgis (known from Almería) and Nesticus luquei (an endemic to northwestern Spain). The shape and arrangement of the median apophysis (Figs 5, 11–12), the embolus (Figs 3–7, 11–12) and the paracymbial processes of male palp (Figs 8–10), plus the location and structure of the spermathecae and vulval glands of the female (Fig 17) are similar in all three species. Nesticus baeticus sp. n.differs more significantly from Nesticus lusitanicus, both in the morphology of the copulatory organs of both sexes.

The new species cannot be assigned to Carpathonesticus, Typhlonesticus or Canarionesticus, and differs from their representatives in having a different shape, ramification and modifications associated to the paracymbium, the general structure and arrangement of the embolous, as well as of the p1 and p2 TTA processes. Yet, the shape and disposition of the spermathecas and vulval glands shows markedly differences.

#### Description of the male

(Holotype). *Coloration:* carapace uniform yellowish. Opisthosoma grayish, with some clearly-marked darker patches. Appendages of the same colour as the carapace, slightly darker around distal segments. Sternum yellowish, slightly paler than the carapace. *Carapace*: approximately circular in dorsal view. Cephalic region not raised but differentiated from the rest of the prosoma. Fovea and thoracic grooves clearly visible. Significantly reduced eyes, more evident in the AM (Fig. 1). Eye size and interocular distances: AM = 0.03; AL = 0.07; PM = 0.06; PL = 0.07; AM - AL = 0.14 AL; AM - AM = 0.03; PM - PL = 0.14; PM - PM = 0.18 PM; PL - AL almost touching. *Opisthosoma*: sub-elliptical in dorsal view. *Appendages*: prolateral margin of the chelicerae with 3 teeth, the two distal ones larger. *Male palp*(Figs 3–7). Paracymbium large (Figs 3–5) with well-developed dorsal and ventral processes (Figs 8–10). Broad, translucent dorsal process with a saw-toothed upper edge (Figs 8–10). Dorsomedial apophysis small and pointed (Fig. 9). Ventral region apically notched (Fig. 10). Short paradistal region, almost conical (Figs 8–9). Distal apophysis long, acuminate and slightly curved (Figs 8–10). Poorly developed MA, reduced to a small fingerlike process fused to the tegulum (Figs 5, 11–12). Conductor absent. TTA with two processes, TTA p1 and TTA p2 (Figs 11–12) (homologous to p1-p6 processes of conductor complex in Huber 1993). TTA p1 is saddle-shaped, longer than wide, slightly curved and serrated in the central area (figs 11, 12). TTA p2 is located in apical position and serves as a conductor of embolus (figs 11–13). Embolus filamanteous with a semicircular course progressively acuminate towards the apex and partially bordering the tegulum (Figs 3–5, 12). *Measurements*: PL: 3.3; PW: 3.1; OL: 3.1; OW: 2.4; total body length = 6.4. Leg I>leg IV>leg II> leg III.

**Table:** 

Leg	coxa	troc.	femur	patella	tibia	meta.	tarsus	total
I	1.1	0.7	9.4	1.4	9.1	9.7	2.9	34.3
II	1.0	0.4	6.9	1.4	6.6	6.9	2.6	25.8
III	0.9	0.6	5.1	1.1	4.0	4.6	1.9	18.2
IV	1.1	0.6	8.3	1.4	6.3	6.6	2.3	26.6

#### Description of the female.

**Figures 14–17.**
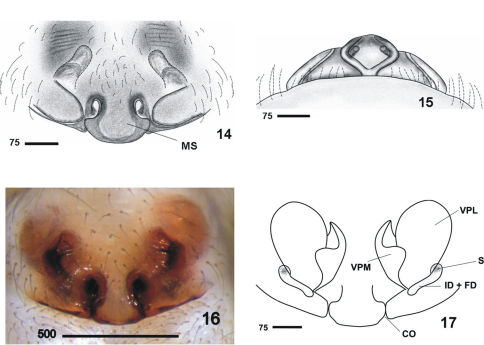
Nesticus baeticus sp. n., female. **14** epigynum (1530) ventral view **15** ditto, caudal view **16** epigynum (1619) ventral view **17** vulva ventral view. Abbreviations: **CO** copulatory orifice **ID + FD** insemination duct + fertilization duct **MS** median septum **S** spermathecae **VPL** vulval pocket lateral **VPM** vulval pocket medial. Scale bar in μm.

All characters as in male except: cephalic region scarcely differentiated, much less marked than in male. Fovea visible and thoracic grooves not clearly marked. *Epigynum and vulva.* Epigynum wide and convex (Figs 14–16). Median septum wide and prominent, caudally projected by a bell-shaped flap. Vulva (Fig. 17, drawing is based on specimen 3812-150) with well-developed lateral pockets divided by a ventral fold in two, the lateral and medial part. *Measurements:* PL: 2.9; PW: 2.9; OL: 3.4; OW: 2.6; total length = 6.3. Leg I>leg IV>leg II> leg III.

**Table:** 

Leg	coxa	troc.	femur	patella	tibia	meta.	tarsus	total
I	1.1	0.7	8.9	1.1	8.9	8.6	3.4	32.7
II	1.0	0.6	8.0	1.1	6.0	6.3	1.4	24.4
III	0.9	0.4	6.3	1.0	3.4	4.0	1.7	17.7
IV	1.3	0.9	7.4	1.0	6.0	6.3	1.7	24.6

#### Distribution.

Nesticus baeticus sp. n. inhabits the karst landscapes of the high part of the Cazorla, Segura and Las Villas Natural Park (NE Jaén, Spain) where it has been found in 8 caves. Most of the material studied comes from the area surrounding the Tranco’s Reservoir, in Hornos, Jaén. The area is calcareous, lush and quite humid, with numerous, medium-sized caves, both horizontal and vertical. The specimens were generally located within the first few meters of the dark zone, their presence reaching towards the cave interiors, which were sampled more intensively.

## Discussion

Specimens and sequences, with corresponding Genbank accession numbers, analyzed in the present study, are listed in Appendix 1. Alignments of two mitochondrial genes and gap scores as presence/absence were merged resulting in a combined data matrix of 950 characters (*cox1*=472, *rrnL*=456 and 22 gap characters). Uncorrected *cox1* genetic divergences among terminal taxa are summarized in Appendix 2. Parsimony analyses of the combined data matrix yielded a single most-parsimonious tree of 705 steps (CI = 73 and RI = 62) (Fig. 1).

The results show that the Iberian species do not constitute a monophyletic group. Nesticus luquei, Nesticus lusitanicus and Nesticus baeticus sp. n. form a clade with high bootstrap support (99%), while Nesticus cellulanus is nested within a clade that also includes Nesticus ionescui, from Romania, and Nesticus eremita, from Croatia. Nesticus obcaecatus is the sister group of the remaining species of the ingroup. This topology, along the high genetic divergences observed between Nesticus obcaecatus, Nesticus cellulanus and the remaining Iberian species suggest the existence of tree independent colonization to the Iberian Peninsula. Preliminary results of a more extensive phylogenetic analysis, including almost all the Mediterranean species of Nesticidae (our work in progress), support this hypothesis.

The morphology of both the male and female copulatory organs of this Iberian group of species (Nesticus luquei, Nesticus lusitanicus, Nesticus murgis and Nesticus baeticus sp. n.) shows important differences as compared to Nesticus cellulanus, the type species of the genus Nesticus, as well as to the Carpathonesticus species. Thus, the absence of paracymbial ramification, the shape and size of the median apophysis, plus clear differences in size and arrangement of the TTA processes constitute the major differences in the males. The number of spermathecae is the most noticeable character in the females.

With regards to Nesticus obcaecatus, significant differences in the shape and structure of the paracymbium, the median apophysis and the TTA (see Ribera 1988), as well as in the shape of the epigynum and the arrangement of the spermathecae (see Fage 1931), indicate that this species is most distantly-related to all the Iberian endemics known to date. On the basis of the aforementioned characters Nesticus obcaecatus seems to be more closely-related to Nesticus idriacus Roewer, 1931 known from the eastern area of the Alps and to Nesticus morisii Brignoli, 1975 known from Italy. Besides, the conformation of the copulatory organs of these three species are very similar to Typhlonesticus absoloni (Kratochvil, 1933) known from Montenegro. Thus, all three species are likely to belong with Typhlonesticus as well. Yet, in order to prove or reject this assumption, a molecular phylogeny of all the Mediterranean Nesticidae is to be performed. Our future research is focused on resolving the phylogeny of the Mediterranean Nesticidae (work in progress) and will include most of the Mediterranean species to test the monophyletic status of current genera.

Nesticus obcaecatus shows highly troglomorphic characters, such as complete depigmentation, reduction of the eye size and their number (only six eyes), and is known from a single cave. These data alongside its phylogenetic uniqueness (basal position and a deep genetic distance from other Iberian congeners) suggest that this species may be considered a relict representative of an old colonization to Iberia, and should be a candidate for protection.

Climatic relict hypothesis assume that adaptation and speciation to caves are mainly driven by climatic factors. The Pleistocene glacial cycles has been identified as de main driver of the evolution of cave-dwelling fauna in the Paleartic (Barr 1968; Vandel 1958, 1964). The uncorrected genetic distances between Nesticus baeticus, Nesticus lusitanicus and Nesticus luquei range between 14.3 and 15.5%. Assuming an average substitution rate for arthropod mitochondrial genes between 2% (DeSalle et al. 1987) to 2.3% (Brower 1994) we can conclude that the origin of these species preceded the Pleistocene glacial cycles and, hence that other climatic or environmental factors may have been responsible for the evolution of these taxa.

## Supplementary Material

XML Treatment for Nesticus baeticus

## Appendix 1

Species included in the cladistic analysis and GenBank accession numbers for the cox1 and *rrnL.* All accession numbers starting with EU are new sequences obtained in the present study.

**Table:**

species	Locality	cox1	rrnL
*Nesticus obcaecatus*	Cueva del Molino de Aso, Valle de Añisclo, Huesca, Spain	EU746428	EU746437
*Nesticus luquei*	Cueva de la Picona, San Pedro de Carmona, Cabuerniga, Cantabria, Spain	EU746430	EU746439
*Nesticus lusitanicus*	Algar de Marradinhas II Concelho de Alcanena, Portugal	EU746429	EU746438
*Nesticus baeticus*	Sima Irene, Hornos, Jaén, Spain	EU746431	EU746440
*Nesticus baeticus*	Sima del Campamento, Hornos, Jaén, Spain	EU746432	EU746441
*Nesticus baeticus*	Sima de los Alhaurinos, Hornos, Jaén, Spain	EU746433	EU746442
*Nesticus ionescui*	Pestera Tismana, Tismana, Romania	EU746434	EU746443
*Nesticus cellulanus*	Manantiales Monte Castro, Sueras, Castellón, Spain.	EU746435	EU746444
*Nuesticus eremita*	Cave Pishurka (=Paganetijeva Pécina), Korcula Is., Croatia.	EU746436	EU746445
“*Nesticus*” X130	China	AY231024	AY230941

## Appendix 2.

Uncorrected genetic distances of *cox 1* gene between terminal taxa analyzed in the present study. Numbers on terminals refers to different localities (see material examined)

**Table:**

	obcaeca	lusitan	luquei	baet5014	baet3860	baet3812	ionescui	cellula	eremita
lusitanicus	0.173								
luquei	0.180	0.143							
baeticus5014	0.197	0.144	0.153						
baeticus3860	0.199	0.143	0.155	0.002					
baeticus3812	0.199	0.143	0.155	0.002	0.000				
ionescui	0.159	0.164	0.170	0.160	0.161	0.161			
cellulanus	0.153	0.168	0.176	0.168	0.169	0.169	0.110		
eremita	0.159	0.155	0.161	0.153	0.151	0.151	0.115	0.117	
NesticusX130	0.169	0.193	0.195	0.204	0.206	0.206	0.174	0.184	0.170
